# Dietary supplementation of laminarin improves the reproductive performance of sows and the growth of suckling piglets

**DOI:** 10.1186/s40104-023-00920-6

**Published:** 2023-09-10

**Authors:** Pengguang Zhang, Guoyuan Jiang, Chenghong Ma, Yubo Wang, Enfa Yan, Linjuan He, Jianxin Guo, Xin Zhang, Jingdong Yin

**Affiliations:** State Key Laboratory of Animal Nutrition and Feeding, College of Animal Science and Technology, China Agricultural University, Beijing, 100193 China

**Keywords:** Fecal microbiota, Laminarin, Milk, Piglets, Reproductive performance, Sows

## Abstract

**Background:**

Maternal nutrition is essential in keeping a highly efficient production system in the pig industry. Laminarin has been shown to improve antioxidant capacity, reduce the inflammatory response, and favor the homeostasis of intestinal microbiota. However, the effect of dietary supplementation of laminarin on the reproductive performance of sows and the growth of suckling offspring remains unknown.

**Methods:**

A total of 40 Landrace × Yorkshire multiparous sows on d 85 of gestation, similar in age, body weight (BW), parity and reproductive performance, were randomly divided into four dietary treatments with 10 sows per treatment, receiving a control diet (basal pregnancy or lactating diets) and a basal diet supplemented with 0.025%, 0.05% and 0.10% laminarin, respectively. The experiment lasted from d 85 of gestation to d 21 of lactation.

**Results:**

Laminarin supplementation linearly increased number born alive per litter (*P* = 0.03), average daily feed intake (ADFI, *P* < 0.01), and total milk yield of sows during the lactation of 1–21 d (*P* = 0.02). Furthermore, maternal laminarin supplementation increased the average daily gain (ADG) of piglets while tending to reduce the culling and death rate before weaning. In addition, alterations to the composition of colostrum and milk, as well as to serum inflammatory cytokines and immunoglobulins of sows were observed. The fecal microbiota profile of sows supported the improvement of reproductive performance in sows and the growth performance in suckling offspring.

**Conclusions:**

Dietary supplementation of laminarin during late pregnancy and lactation could significantly improve reproductive performance of sows and growth performance of piglets.

**Graphical Abstract:**

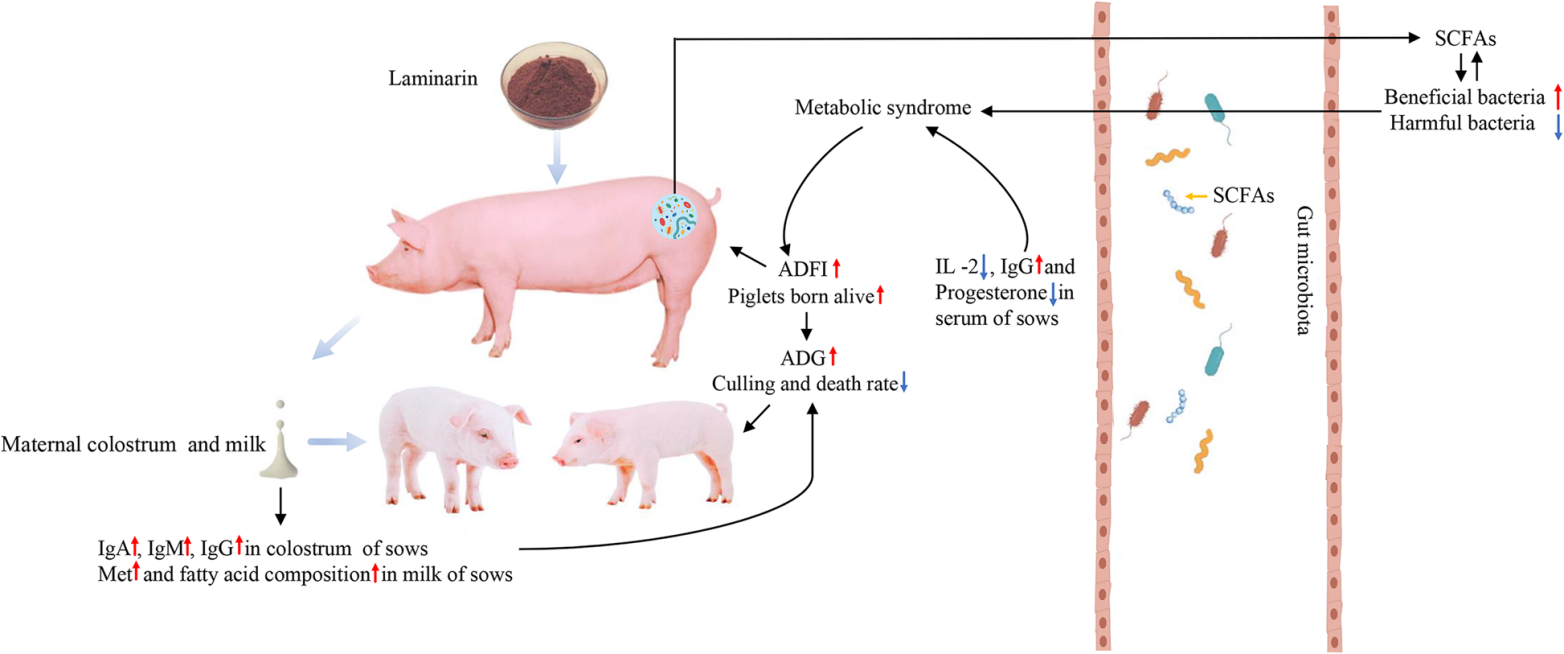

**Supplementary Information:**

The online version contains supplementary material available at 10.1186/s40104-023-00920-6.

## Background

Late pregnancy is a critical period for rapid fetal development, and maternal nutrition plays a vital role not only in the growth but in the development of the immune system of suckling offspring [1]. During late pregnancy, sows undergo dramatic changes in physiology, metabolism and immunity due to rapid fetal development [2]. Minimizing inflammatory response and ensuring normal metabolism and immunity of sows during late pregnancy is essential for the reproductive performance of sows and the development of intestinal microbiota [3]. Further, the intestinal microbiota plays a key role in nutritional metabolism, immune development, resistance to pathogens, and the pathogenesis of many chronic diseases in the host [3, 4]. The intestinal microbiota of sows is involved in the metabolism of pregnant sows and can change significantly during gestation [5]. The maternal microbial communities are transferred to the newborn and shape the intestinal microbiota of suckling offspring [6]. Therefore, special attention should be paid to the role of intestinal microbiota communities in maternal health and suckling offspring growth and development in the process of regulating maternal diets.

Maternal milk can not only provide nutrition for the baby, but also ensure the health of the newborn [7]. In addition, colostrum contains abundant immunoglobulins. As the immune system is immature, newborn piglets cannot cope with the invasion of pathogens. Immunoglobulin G (IgG), IgM and IgA are crucial for protecting the health of offspring [8]. The abundant immunoglobulins in colostrum and fatty acids in milk are key for newborn and lactating piglets to keep good health and growth, which are strongly affected by the maternal nutritional status. As a prebiotic, laminarin is mainly present in the cell wall matrix, intercellular space and secreted mucus of brown algae such as kelp, *Undaria pinnatifida*, giant algae and hornwort [9, 10], it is known that its main active ingredient is fucoidan, a unique naturally active polysaccharide of sulfate in brown algae [10]. Laminarin is widely used in food and pharmaceutical industries because of its outstanding anti-tumor, anti-mutagenic activities, and anti-inflammatory as well as antioxidant properties [11]. Dietary laminarin supplementation has been shown to benefit gut health and microbiota composition, and reduce the use of antimicrobial agents in piglet diets [12]. The maternal diet during fetal and postnatal development is a key determinant of the immediate health and offspring health in later life, and the maternal intake of prebiotics and antioxidants is a good way to ensure normal growth and development of offspring. The effects of laminarin on reproductive performance of sows and growth performance of suckling offspring have not been reported. Moreover, the ingestion of marine products such as algae has been shown to improve the composition of milk [13]. However, the effect of laminarin on milk composition remains unknown yet. Given the beneficial properties of laminarin, we hypothesize that dietary laminarin supplementation in sow's diet might improve sow health and reproductive performance, and improve the growth of lactating piglets as well. Therefore, the study aimed to evaluate the effects of dietary laminarin supplementation on reproductive performance, and nutrition composition of colostrum and milk, as well as fecal microbiota of sows.

## Methods

### Animals and experimental design

All animal experiments were approved by the Animal Care and Use Committee of China Agricultural University (approval number: SKLAB-2011–04-03). A total of 40 Landrace × Yorkshire multiparous sows at d 85 of gestation with similar age, body weight, parity and reproductive performance were randomly divided into four dietary treatments, with 10 sows in each treatment. Sows were fed control diets (basal pregnancy or lactating sow diet) and basal diets supplemented with 0.025%, 0.05% and 0.10% laminarin, respectively. In this experiment, laminarin is provided by Rongcheng Hongde Marine Biotechnology Co., Ltd. (Weihai, Shandong Province, China). The main components are various bioactive components such as fucoidan. The specific components were shown in Table 1. The basal diets of sows during late pregnancy and lactation were formulated according to NRC (2012) [14]. The ingredients and nutrient compositions of the basal diets were shown in Table 2. The experiment lasted from d 85 of gestation to d 21 of lactation.

**Table 1:** Chemical composition of laminarin

Chemical composition	Content
Fucoidan (as fucose), %	16.50
Sulfate, %	2.80
Total sugar (as glucose), %	7.14
Mannitol, %	16.00
Alginic acid, %	1.80
K, mg/kg	1.64 × 10^5^
P, mg/kg	1.67 × 10^4^
Organic matter (dry basis), %	15.27
Water insoluble matter, %	2.70
Protein, %	7.48
Crude ash, %	50.80
Ribose, mg/kg	628.18
Rhamnose, mg/kg	1,153.89
Glucuronic acid, mg/kg	6,523.74
Galacturonic acid, mg/kg	336.55
Glucose, mg/kg	3,367.88
Galactose, mg/kg	6,631.72
Xylose, mg/kg	1,687.09
Fucose, mg/kg	9,225.22
Acid detergent fiber, %	0.50
Neutral detergent fiber, %	3.60

**Table 2:** Ingredients and nutrient levels of basal diets of sows during late gestation and lactation (as-fed basis)

Items	Late gestation	Lactation
Ingredients, %		
Corn	59.00	55.00
Extruded corn	2.00	5.00
Soybean meal	14.00	17.00
Extruded soybean	2.00	3.00
Fermented soybean meal	——	2.00
Wheat bran	16.00	6.00
Fish meal	3.00	5.00
Fatty powder^a^	——	3.00
Premix^b^	4.00	4.00
Nutrient composition (calculated value)^c^, %		
DE, Mcal/kg	3.15	3.30
CP	16.24	18.04
Ca	0.95	0.94
TP	0.63	0.71
SID Lys	0.70	0.93
SID Met	0.24	0.28
SID Met + Cys	0.53	0.54
SID Val	0.61	0.69
SID Thr	0.49	0.55
SID Trp	0.16	0.17

^a^The main ingredients of fatty powder are concentrated soybean phospholipid oil, puffed corn and emulsifier

^b^Premix provided per kilogram of complete feed: vitamin A, 240–300 KIU; vitamin D_3_, 50–125 KIU; vitamin E, 500 IU; vitamin K_3_, 45 mg; vitamin B_1_, 50 mg; vitamin B_2_, 150 mg; vitamin B_6_, 100 mg; vitamin B_12_, 0.5 mg; niacin, 650 mg; pantothenic acid, 450 mg; folic acid, 80 mg; biotin, 10 mg; Fe, 2.4–18 g; Cu, 0.2–0.62 g; Zn, 1–2.5 g; Mn, 0.5–2 g; I, 10–50 mg; Se, 5–12.5 mg; calcium, 80 g; phosphorus, 60 g; choline chloride, 10 mg; water, 10%

^c^Nutrient composition: calculated chemical concentrations using values for feed ingredients from NRC (2012) [14]

### Feeding management

This experiment was conducted in the Fengning Swine Research Unit of China Agricultural University (Academician Workstation in Chengde Jiuyun Agricultural and Livestock Co., Ltd., Hebei, China). Each treatment included 10 replicated pens, each of which housed one sow. Sows had free access to water throughout the experiment. From d 85 of gestation to parturition, all sows were fed 3.0 kg/d diet and fed three times daily at 05:30, 10:00 and 15:00. On d 107 of gestation, sows were moved into farrowing rooms with environmental control systems (22–26 °C) and housed in individual farrowing pen (2.1 m × 1.5 m). On the parturition day, sows were fed 0.5 kg of lactation diet and the ration was gradually increased by 1.0 kg/d until free access to feed. From d 2 after delivery, lactating sows were fed three times a day. We weighed and removed uneaten feed every morning and recorded feed intake by subtracting uneaten feed from the feed provided. Piglets were not provided with feed during lactation. Other routine management and immunization procedures were carried out according to pig farm breeding procedures, including tail cutting, tooth cutting, iron supplementation, castration, etc. The litter size at birth, number born alive per litter and number of healthy piglets were recorded. Piglets with the birth body weight more than 1.0 kg were considered healthy. The BW of each piglet and litter weight were recorded at birth. Additionally, feed intake of each sow was recorded during lactation to calculate ADFI. The BW of sows at d 109 of gestation and weaning was recorded to calculate the BW loss of sows during lactation. The backfat (BF) thickness of sows on d 85 of pregnancy was measured at the P1 point on the left side of the back of sows (6.5 cm from the last rib to the midline of the back) using a backfat meter (Italy, Mylab Touch Vet). The BF was used to evaluate the body condition and grouping of sows. The BF at d 1 of lactation and weaning was measured. Within 24 h after delivery, newborn piglets were cross-fostered within each treatment group to about 13 piglets per litter. The number, weight and litter weight of piglets per litter after cross-fostering and at d 21 of lactation were recorded. The culling and death rate, ADG and daily litter gain of piglets during lactation were calculated. The total milk yield of an individual sow during 21 d of lactation was calculated on the basis of the piglet ADG and litter size using the following equation: Total milk yield = piglet ADG × litter size × lactation days × 4 [15].

### Sample collection and preparation

Ten mL blood samples from ear vein were collected from all sows at farrowing, and centrifuged at 3,000 × *g* for 15 min at 4 °C to obtain serum. About 20 mL of colostrum was collected from the third and fourth pairs of nipples on the same side of the sows immediately after delivery to detect the nutritional components and immunoglobulin content in colostrum. Oxytocin was not used during the collection of colostrum. On d 14 of the sows' lactation, about 20 mL of milk was collected using gentle stripping of teats due to the stimulation by oxytocin injections. Serum, colostrum and milk samples were stored at −80 °C for further analysis. Fresh feces (*n* = 8) were collected from the rectum of sows on d 105 of gestation and stored at −80 °C for subsequent analysis.

### Analysis of serum indicators

The levels of tumor necrosis factor-α (TNF-α), interferon-γ (IFN-γ), interleukin-6 (IL-6), interleukin-2 (IL-2), interleukin-10 (IL-10), IgA, IgG, and IgM in serum of sows were determined by commercial kits (Nanjing Jiancheng Bioengineering Institute, Jiangsu, China) according to the manufacturer’s instructions. The contents of estradiol, progesterone, leptin, glucose, insulin, and pituitary prolactin in the serum of sows were measured by commercial kits (Nanjing Jiancheng Bioengineering Institute, Jiangsu, China) as referred by the manufacturer’s instructions.

### Colostrum and milk composition

Frozen colostrum and milk samples were thawed. Fat, protein, lactose, density, ash content and non-fat solid were measured by Milk Composition Analyzer (Milk-YwayCP2, Beijing, China). Concentrations of immunoglobulins including IgA, IgG and IgM were measured by ELISA kits (Nanjing Jiancheng Bioengineering Institute, Jiangsu, China) in colostrum and milk. The fatty acid composition of milk was determined by Agilent 6890 gas chromatography analyzer. Two mL of milk sample was taken and 8 mL of chloroform–methanol solution (2:1) was added. After being fully mixed in the test tube, it was transferred to a centrifuge tube and centrifuged at 3,000 r/min for 10 min. The underlying fluid was dried at indoor temperature in a 50-mL round-bottomed flask, and then methyl esterified. After methyl esterification, it was determined by gas chromatography. The determination conditions were as follows: chromatographic column DB-23 (60 m × 0.25 mm id × 0.25 μm), inlet temperature of 260 °C, pre-column pressure of 15 psi, injection volume of 1 μL, shitter ratio of 30:1, FID detector at 280 °C, H_2_ flow rate was 30 mL/min and air flow rate was 400 mL/min. Analysis method of the amino acid composition of milk was as follows: 1 mL sample was taken and added to the hydrolysis tube, adding 15 mL HCl with a concentration of 6 mol/L, vacuum sealing, hydrolysis at 100 ± 1 °C for 24 h, filtration, constant volume to 50 mL, taking 1 mL of filtrate at 20 °C under reduced pressure and dry, then 0.02 mol/L HCl for constant volume, and finally loading sample, using amino acid analyzer ( Hitachi 835–50, Japan) for analysis.

### Fecal SCFAs in sows

Fecal samples were weighed 1 g, added with 8 mL deionized water, dissolved, homogenized, centrifuged at 5,000 × *g* for 10 min, and the supernatant was taken. The supernatant was diluted 50-fold and filtered through a 0.22-μm filter (Millipore, Bedford, UK). The filtrate (25 μL) was stored in a 2-mL spiral cap bottle, and then the contents of SCFAs were determined by an ion chromatography system (Thermo Fisher Scientific, Wilmington, DE, USA).

### DNA extraction, 16S rRNA sequencing and data analysis

Microbial genomic DNA was extracted from fecal samples, final DNA concentration was determined using a NanoDrop 2000 UV–Vis spectrophotometer (Thermo Fisher Scientific, Wilmington, DE, USA), and DNA quality was assessed by 1% agarose gel electrophoresis. The V3 + V4 regions of 16S rRNA genes were amplified by PCR with primer pairs (Forward: 5′-ACTCCTACGGGAGGCAGCAG-3′, Reverse: 5′-GGACTACHVGGGTWTCTAAT-3′) to produce the fragments of about 500 bp using the TransStart Fastpfu DNA Polymerases (TransGen Biotech, Beijing, China). The PCR product was extracted from 2% agarose gel and purified using the AxyPrep DNA Gel Extraction Kit (Axygen Biosciences, Union City, USA). Purified amplicons were pooled in equimolar amounts and paired-end sequenced on an Illumina MiSeq PE300 platform/NovaSeq PE250 platform (Illumina, San Diego, USA) according to the standard protocols by Majorbio Bio-Pharm Technology Co., Ltd. (Shanghai, China). To form operational taxonomic units (OTUs), all raw sequences were filtered, denoised, merged, and removed non-chimerics by the DADA2 plug-in in Qiime2 software (https://qiime2.org/). The cluster analysis and taxonomic analysis were then performed for all OTUs. The representative sequences of OTUs were compared with the Silva Release 138 database to obtain the annotation information of species.

### Statistical analysis

Results were expressed as means ± SEM, and analyzed by the unpaired two-tailed Student’s *t*-test or the linear and quadratic regression analyses of SAS (v.9.2, SAS Institute, USA). Linear and quadratic regression analyses were employed to evaluate effects of laminarin dose on reproductive performance, growth performance of piglets, and serum parameters of sows. Because of significant effect of 0.10% dietary laminarin supplementation on most cases, we subsequently investigated the effect of laminarin on the nutritional composition of colostrum and milk by comparing the control group and 0.10% laminarin supplementation. The *t*-test was used to test whether the difference was significant between control and laminarin groups. For the productive performance of sows, each sow was treated as an experimental unit. For the growth performance of piglets, each litter was treated as an experimental unit. Spearman's correlation was used to evaluate the relationships among key parameters. Value of *P* < 0.05 was considered significant, and 0.05 ≤ *P* ≤ 0.10 was considered to have a trend.

## Results

### Dietary laminarin supplementation can improve the performance of sows and suckling piglets

A total of 40 sows were selected at the beginning of the experiment, but 4 sows were excluded due to illness and lameness. The final results were 9, 9, 8, and 10 sows in the control group, 0.025% laminarin group, 0.05% laminarin group, and 0.10% laminarin group, respectively. As shown in Table 3, the addition of laminarin to the diet during late pregnancy-lactation linearly increased number born alive per litter (*P* = 0.03) and ADFI during the lactation (*P* < 0.01). Meanwhile, maternal supplementation of laminarin linearly increased ADG of piglets (*P* = 0.04, Table 3), and showed a tendency to reduce the culling and death rate of piglets (*P* = 0.09). What’s more, dietary supplementation of laminarin improved the milk yield of sows during lactation (*P* = 0.02, Table 3).

**Table 3:** Effects of the dietary laminarin supplementation on the performance of the sows and piglets (*n* = 8–10)

Items	Laminarin levels, %				SEM	*P-*value	
	**0**	**0.025**	**0.05**	**0.10**		**Linear**	**Quadratic**
Sows							
Average of parity, *n*	6.89	7.22	8.00	6.80	0.27	0.91	0.13
Average daily feed intake, kg/d							
1^st^ week of lactation	3.79	3.64	3.78	4.41	0.15	0.08	0.30
2^nd^ week of lactation	6.57	7.12	7.48	7.73	0.19	0.03	0.46
3^rd^ week of lactation	7.08	6.69	7.28	7.87	0.17	0.03	0.34
1 to 21 d of lactation	5.96	5.94	6.18	6.57	0.09	< 0.01	0.52
Total milk yield^a^, kg	159.85	198.72	188.90	218.96	8.60	0.02	0.66
BW, kg							
Pregnancy for 109 d	282.00	282.89	277.20	286.00	3.43	0.72	0.57
After weaning	270.00	271.44	272.60	273.67	3.85	0.73	0.94
BW loss	12.00	11.44	4.60	12.33	2.07	0.95	0.28
Backfat thickness, mm							
G85	17.80	19.33	16.22	18.90	0.64	0.80	0.46
L1	20.88	21.50	22.00	22.63	0.67	0.36	0.88
L21	15.63	14.75	16.33	17.13	0.45	0.11	0.64
BF loss from L1 to L21	5.25	6.75	5.67	5.50	0.50	0.88	0.55
Piglets							
Litter size at birth, *n*	14.78	14.44	15.56	16.22	0.48	0.21	0.85
Number born alive per litter, *n*	13.22	13.67	14.13	15.28	0.37	0.03	0.89
Number of healthy piglets, *n*	12.00	12.33	12.38	11.90	0.33	0.77	0.85
Average birth weight, kg	1.41	1.36	1.25	1.34	0.03	0.27	0.07
Litter weight at birth, kg	19.06	19.01	17.65	19.70	0.49	0.70	0.25
Average weight after cross-fostering, kg	1.75	1.58	1.54	1.65	0.05	0.63	0.20
Average weaning weight, kg	5.79	6.16	6.03	6.40	0.13	0.13	0.91
Average daily gain (ADG), kg/d	0.19	0.22	0.21	0.23	0.01	0.04	0.45
Litter weight after cross-fostering, kg	21.26	20.56	18.95	22.11	0.64	0.62	0.13
Litter weight at weaning, kg	64.77	68.20	64.38	72.38	2.47	0.33	0.68
Daily litter gain during lactation, kg/d	2.07	2.27	2.16	2.40	0.10	0.34	0.97
Litter size after cross-fostering, *n*	12.67	13.00	12.71	12.13	0.17	0.15	0.30
Weaned piglets, *n*	10.67	11.11	11.00	11.63	0.27	0.25	0.96
Culling and death rate, %	15.77	14.48	13.46	5.63	2.16	0.09	0.62

^a^Total milk yield = piglet ADG × litter size × lactation days × 4; values are means with SEM; healthy piglets: birth weight ≥ 1 kg

### Concentrations of inflammatory factors and immunoglobulins in the serum of sows

As shown in Table 4, dietary supplementation of laminarin decreased the concentration of IL-2 (*P* = 0.03) but increased IgG concentration (*P* = 0.01) in serum of sows on the day of parturition. Laminarin supplementation did not alter the concentrations of TNF-α, IFN-γ, IL-6, IL-10, IgA and IgM.

**Table 4:** Effects of dietary laminarin on inflammatory cytokine levels and immunoglobulin concentrations in serum of sows (*n* = 8–10)

Items	Laminarin levels, %				SEM	*P-*value	
	**0**	**0.025**	**0.05**	**0.10**		**Linear**	**Quadratic**
TNF-α, pg/mL	52.34	51.39	53.73	52.43	1.51	0.89	0.86
IFN-γ, pg/mL	37.49	40.75	39.43	34.82	2.03	0.53	0.43
IL-6, pg/mL	140.36	126.10	125.06	126.16	4.12	0.30	0.30
IL-10, pg/mL	20.48	18.74	18.38	17.13	0.74	0.13	0.71
IL-2, pg/mL	280.02	223.54	223.82	209.25	10.51	0.03	0.21
IgA, g/L	2.39	2.70	2.73	2.69	0.11	0.38	0.38
IgG, g/L	16.83	21.06	23.56	23.10	0.89	0.01	0.05
IgM, g/L	1.91	2.37	2.27	2.15	0.09	0.57	0.12

Values are means with SEM

### Concentrations of endocrine hormones, insulin and glucose in the serum of sows

As shown in Table S1, dietary supplementation with laminarin reduced the concentration of progesterone in sows' serum on the day of parturition (*P* < 0.05), but there were no differences in concentrations of estradiol, leptin, insulin, glucose and prolactin.

### Contents of conventional nutrients and immunoglobulins in colostrum and milk of sows

In the present study, according to the above results, we focused on the role of laminarin in the composition of colostrum and milk by employing the supplementation of 0.10% laminarin in diets, named laminarin group in the subsequent study.

As shown in Table 5, dietary supplementation of laminarin did not alter the conventional nutrients content in colostrum of sows, with increasing the concentrations of IgG and IgM in colostrum (*P* = 0.03) and having a tendency to increase IgA in colostrum (*P* = 0.06). As shown in Table S2, the contents of fat, protein, lactose and non-fat solids in milk were not affected by dietary supplementation of laminarin. Likewise, immunoglobulin concentrations in milk were not altered.

**Table 5:** Effects of dietary laminarin supplementation on nutrient composition and immunoglobulin content in colostrum of sows (*n* = 9)

**Items**	**Laminarin levels, %**		**SEM**	***P*-value**
	**0**	**0.10**		
Nutrients, %				
Fat	4.00	4.29	0.25	0.57
Lactose	8.46	8.29	0.23	0.72
Protein	12.69	12.43	0.35	0.72
Non-fat solid	22.30	22.37	0.44	0.94
Density (20 °C), g/cm^3^	1.08	1.07	0.03	0.83
Ash content, %	1.89	1.85	0.05	0.71
Immunoglobulin, g/L				
IgA	6.29	8.28	0.53	0.06
IgG	49.20	61.08	2.80	0.03
IgM	5.98	7.36	0.28	0.03

Values are means with SEM

### Amino acid composition and fatty acid composition in the milk of sows

As shown in Table S3, supplementation with laminarin tended to increase methionine content in milk of sows compared to the control group (*P* = 0.07), although the content of other amino acids did not change. Interestingly, the contents of capric acid (C10:0), myristic acid (C14:0), tetradecenoylcarnitine (C14:1), palmitoleic acid (C16:1), eicosapentaenoic acid (EPA, C20:5n3) in milk were increased in laminarin-fed sows compared with those of the control group (*P* ≤ 0.05, Table 6).

**Table 6:** Effects of dietary laminarin supplementation on fatty acid composition in milk of sows (*n* = 9), %

**Items**	**Laminarin levels, %**		**SEM**	***P-*value**
	**0**	**0.10**		
C6:0	0.02	0.03	0.001	0.28
C8:0	0.03	0.03	0.001	0.53
C10:0	0.18	0.22	0.010	0.02
C12:0	0.86	0.81	0.040	0.58
C14:0	3.69	4.13	0.117	0.03
C14:1	0.29	0.35	0.015	0.04
C15:0	0.11	0.11	0.004	0.37
C16:0	33.36	33.77	0.628	0.71
C16:1	10.98	12.16	0.365	0.05
C17:0	0.18	0.18	0.010	0.80
C18:0	3.95	3.80	0.123	0.51
C18:1n9c	23.02	24.77	0.817	0.34
C18:2n6c	18.03	17.71	0.470	0.75
C18:3n3	1.43	1.47	0.051	0.72
C20:0	0.12	0.12	0.003	0.42
C20:1	0.19	0.26	0.019	0.12
C21:0	0.29	0.32	0.016	0.42
C20:3n6	0.11	0.11	0.006	0.72
C20:4n6	0.45	0.44	0.017	0.77
C20:3n3	0.08	0.08	0.003	0.85
C22:0	0.14	0.14	0.005	0.50
C20:5n3	0.20	0.24	0.009	0.04
C22:1n9	0.05	0.05	0.003	0.22
C23:0	0.06	0.06	0.005	0.88
C22:2	0.04	0.04	0.002	0.47
C24:0	0.17	0.15	0.009	0.56
C24:1	0.11	0.12	0.005	0.73
C22:6n3	0.36	0.39	0.022	0.47

Values are means with SEM

### Laminarin consumption affected fecal microbiota of sows

**Fig. 1:**
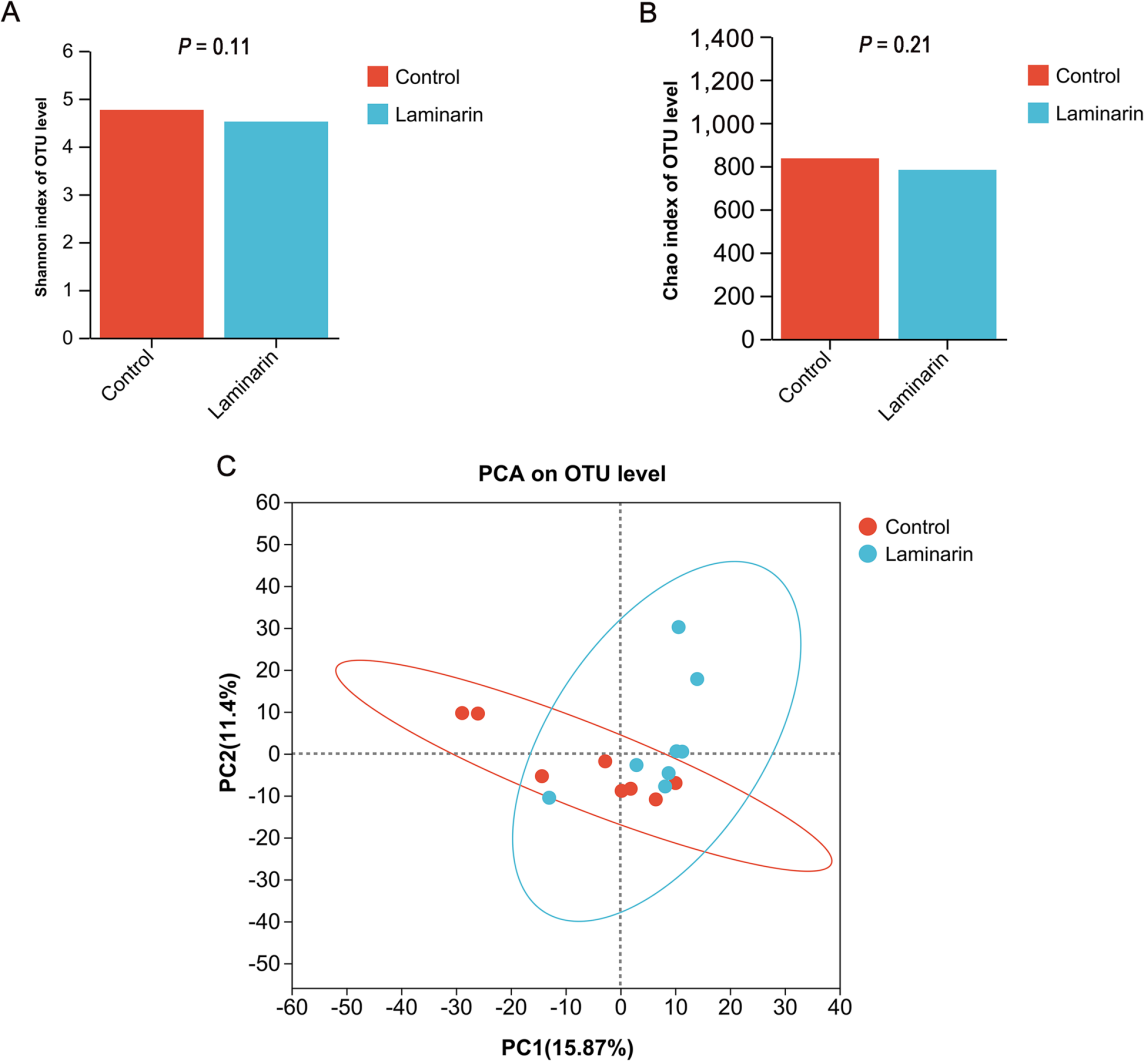
Diversity and richness of bacterial communities. **A** and** B** Alpha diversity metrics. Shannon (**A**) and Chao (**B**) indexes for fecal microbiota of sows. **C** Plot of principal coordinate analysis. A shorter distance between the sample points denotes greater similarity of the bacteria (*n* = 8 for each group)

**Fig. 2:**
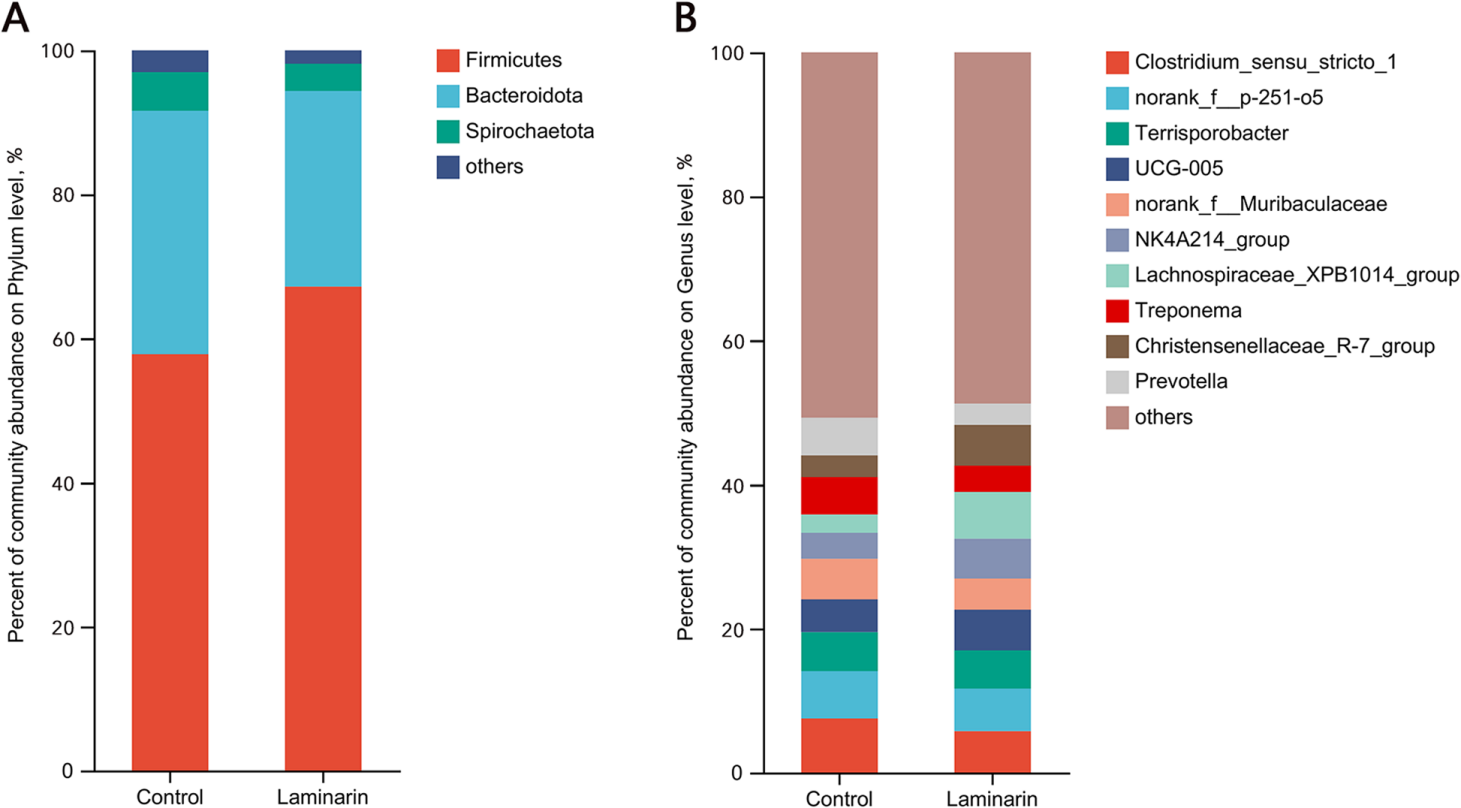
Relative abundance of fecal microbiota of sows at the phylum (**A**) and genus (**B**) levels in the two groups (*n* = 8 for each group)

Dietary laminarin supplementation did not significantly affect both α- and β-diversity indexes of microbiota in feces of sows (Fig. 1A–C). 16S rRNA analysis showed that Firmicutes, Bacteroidota, and Spirochaetota were dominant in feces at the phylum level (Fig. 2A). At the genus level, *Clostridium_sensu_stricto_1* was the most dominant (Fig. 2B).

**Fig. 3:**
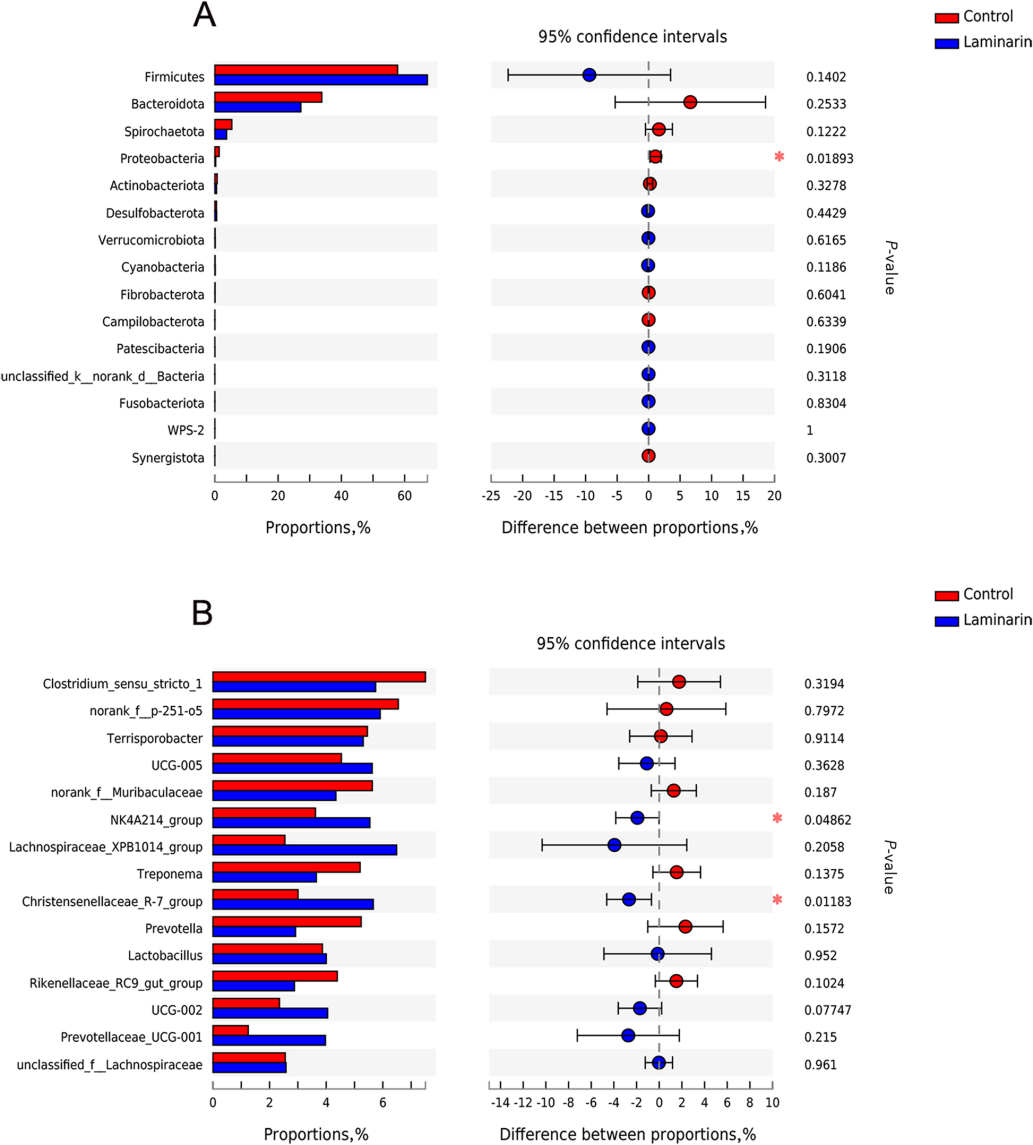
Effects of dietary laminarin supplementation on fecal microbiota of sows. Differences in fecal microbiota of sows between the two groups at phylum level (**A**) and at genus level (**B**) (*n* = 8 for each group). ^*^0.01 < *P* ≤ 0.05, ^**^P < 0.01

In addition, at the phylum level, the relative abundance of Proteobacteria was lower in the laminarin group than in the control group (*P* < 0.05, Fig. 3A). At the genus level, the relative abundance of *Christensenellaceae_R-7_group* and *NK4A214_group* were higher in the laminarin group when compared to the control group (*P* < 0.05, Fig. 3B).

**Fig. 4:**
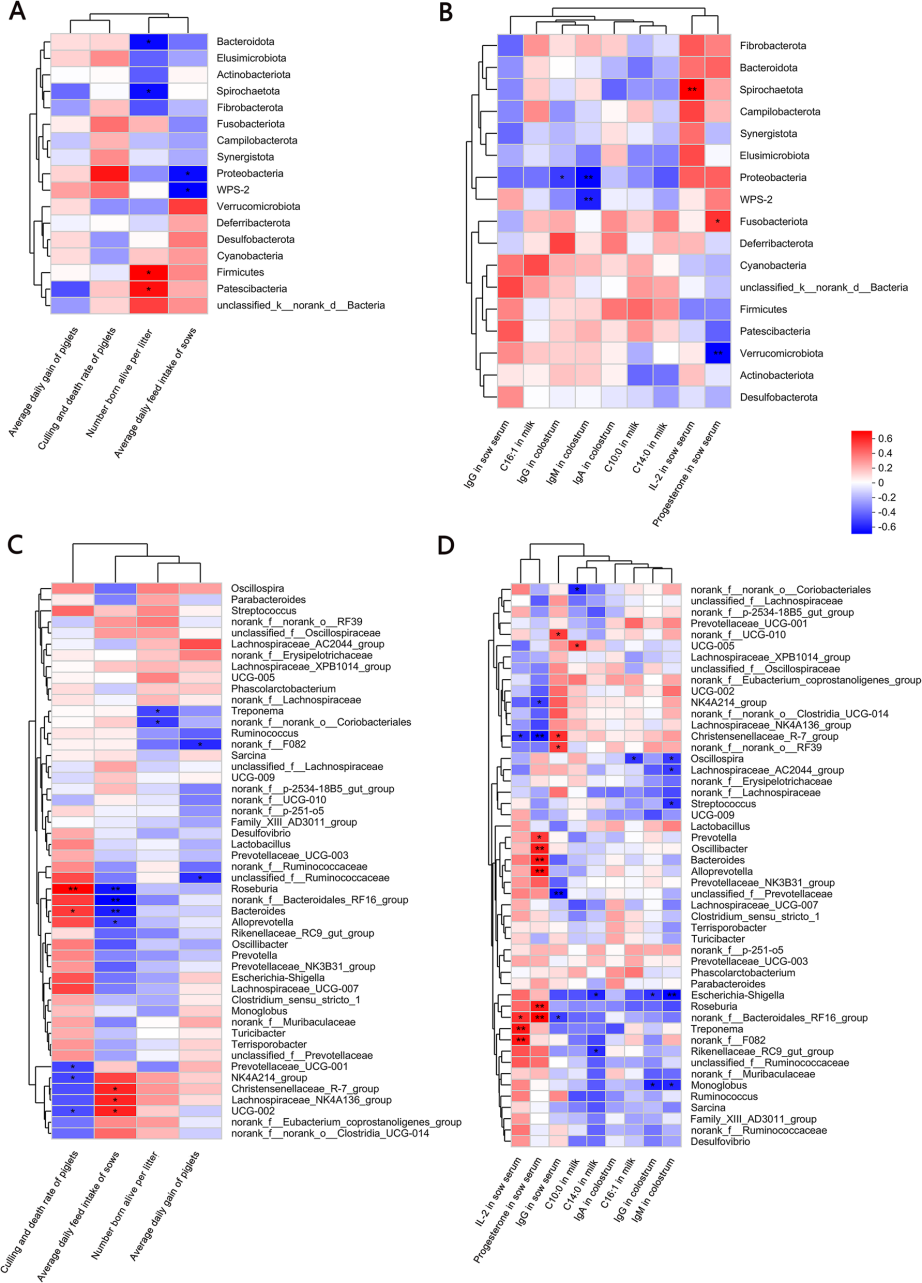
Correlation of fecal microbiota of sows with the parameters of performance, serum, colostrum and milk of sows. **A** Spearman correlation analysis between major phyla and sows' performance. **B **Spearman correlation analysis between major phyla and parameters of serum, colostrum, and milk of sows. **C** Spearman correlation analysis between major genera and sows' performance. **D** Spearman correlation analysis between major genera and parameters of serum, colostrum and milk of sows (*n* = 8 for each group). ^*^0.01 < *P* ≤ 0.05, ^**^P < 0.01

The Spearman’s correlation analysis showed that Proteobacteria was negatively correlated with ADFI, IgM and IgG in colostrum of sows during lactation (*P* < 0.05, Fig. 4A, B), *NK4A214_group* was negatively correlated with culling and death rate of piglets, and serum progesterone level in sows (*P* < 0.05, Fig. 4C, D). *Christensenellaceae_R-7_group* was negatively correlated with contents of IL-2 and progesterone in the serum of sows (*P* < 0.05, Fig. 4D). *Christensenellaceae_R-7_group* was positively correlated with the ADFI of sows and IgG content in the serum (*P* < 0.05, Fig. 4C, D).

**Fig. 5:**
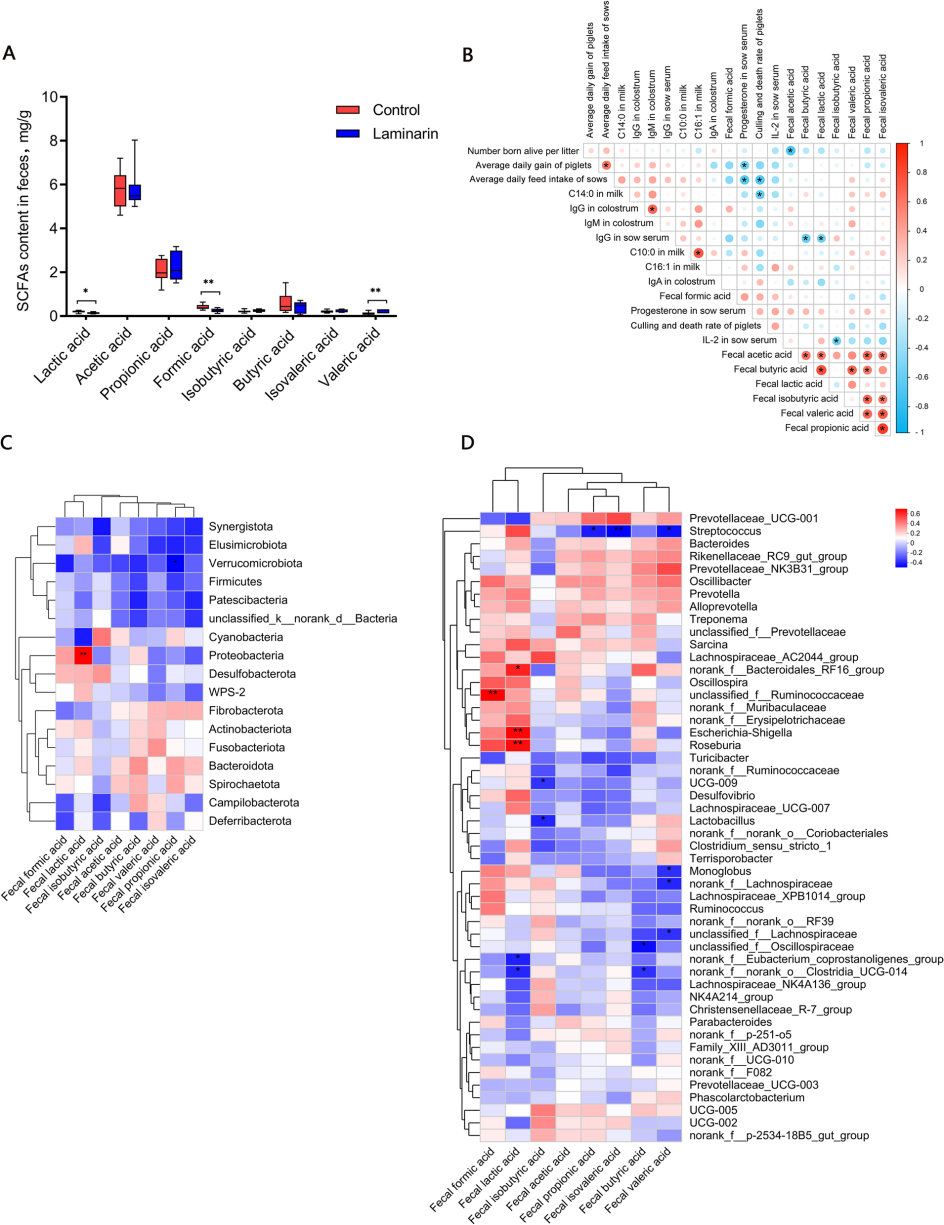
Effects of dietary laminarin supplementation on SCFAs in feces of sows. **A** Effects of dietary laminarin supplementation on the concentrations of SCFAs in feces of sows (mg/g), **B** Correlation among parameters by Spearman correlation analysis, **C** Correlation between gut microbiota and SCFAs in feces of sows by Spearman correlation analysis at phylum level, **D** Correlation between gut microbiota and SCFAs in feces of sows by Spearman correlation analysis at genera level (*n* = 8 for each group). ^*^0.01 < *P* ≤ 0.05, ^**^P < 0.01. Red indicates a positive correlation; blue indicates a negative correlation

As shown in Fig. 5, the contents of lactic acid (*P* < 0.05) and formic acid (*P* < 0.01, Fig. 5A) in the feces of laminarin-fed sows were lower than those of the control sows, while valeric acid content was higher than that of the control (*P* < 0.01). There were no significant differences in the contents of propionic acid, butyric acid, isobutyric acid, and isovaleric acid.

In addition, multiple correlation analyses showed that number born alive per litter was negatively correlated with acetic acid content in the feces of sows (*P* < 0.05, Fig. 5B). During the period of lactation, the ADG of piglets was positively correlated with the ADFI of sows (*P* < 0.05, Fig. 5B).

The culling and death rate of piglets was negatively correlated with C14:0 content of the milk and ADFI of sows during lactation (*P* < 0.05, Fig. 5B). IgG content in colostrum was positively correlated with IgM content in colostrum (*P* < 0.05, Fig. 5B), and IgG in the serum of sows was negatively correlated with the fecal contents of butyric acid and lactic acid (*P* < 0.05, Fig. 5B). C10:0 content was positively correlated with C16:1 content in milk (*P* < 0.05, Fig. 5B), and IL-2 content of serum in sows was negatively correlated with fecal contents of isobutyric acid in sows (*P* < 0.05, Fig. 5B). At the phylum level, Proteobacteria in feces of sows was positively correlated with fecal contents of lactic acid (*P* < 0.05, Fig. 5C).

## Discussion

In intensive pig farming, sows usually suffer long-term stress, resulting in disturbed immune function and damaged reproductive performance of sows [16]. It has been reported that laminarin can inhibit the inflammatory response and improve immune status [17]. However, whether laminarin has effects on the composition of colostrum and milk is not known. In this study, we found that laminarin supplementation improved number born alive per litter, and increased ADFI and milk yield of sows during the lactation. Besides, laminarin supplementation also improved ADG and reduced culling and death rate of suckling piglets.

Previous study has shown that interleukins are involved in the immune process of the body and that the representative IL-2 is high in the period of illness of the body [18]. Under stress or infection, the immune activation of sows leads to the activation of pro-inflammatory cytokines including IL-2 [19]. IgG plays the main immune role, and can resist the invasion of a variety of bacteria and toxins, so as to prevent the body from infection [20]. In this study, IgG content in serum was increased while IL-2 content decreased in laminarin-fed sows compared with those in sows of control group, suggesting that dietary supplementation of laminarin might improve health status of sows.

Previous studies have shown that the quality of colostrum and milk is positively correlated with the increase in body weight gain of offspring during lactation and negatively correlated with mortality of lactation [21]. In this study, it showed that laminarin intake during lactation improved the quality of colostrum and milk in sows. In particular, the contents of IgG, IgM and Ig A in colostrum, as well as the contents of C10:0, C14:0, C14:1, C16:1 and C20:5n3 in milk were increased by laminarin intake. Considering the immature immune system of newborns, sufficient amount of IgA, IgG and IgM in the colostrum is critical to guarantee the survival and healthy growth of neonatal piglets. Immunoglobulin provides the first line of defense on the mucosal surface of newborn piglets against pathogens and viruses [22], reducing the risk of diarrhea, respiratory infections and inflammatory bowel disease [23]. For example, IgG confers passive immunity to newborns and plays a leading role in combating pathogens [24]. Moreover, maternal milk contains all the fatty acids that the body needs [25]. Especially, the presence of n3-PUFAs in maternal milk is essential for the normal development of newborns because n3-PUFAs play an important role in the full development of the brain, retina and other organs [26]. In addition, it has been well indicated that ingestions of C10:0, C14:1 and C16:1 are positively correlated with the growth of infants [27]. Medium-chain fatty acids enhance the intrinsic respiratory capacity of mitochondria without increasing oxidative stress, and these effects potentially contribute to the beneficial metabolic effects of medium-chain fatty acids [28]. The intake of marine products such as algae can increase the levels of monounsaturated fatty acids and polyunsaturated fatty acids in maternal milk [13]. Regarding the beneficial effects of fatty acids from milk on infants, it has been shown that monounsaturated and polyunsaturated fatty acids play an important role in immune regulation and lipid metabolism and are essential for the development of the immune system and the prevention of disease in the offspring [29].

Milk production of sows is another essential factor influencing growth of suckling piglets [30]. Compared with the control group, milk yield significantly increased from 159.85 to 218.96 kg with the supplementation of an extra 0.10% laminarin in the basal diet, which was a 37% increase. Accordingly, the ADG of suckling piglets was significantly increased in linearity by increasing maternal laminarin intake. It is believed that feed intake has a significant impact on how well the sows perform overall. Milk production in sows generally increases with increasing feed intake during lactation [31, 32]. Likewise, in this study, we also observed that the milk yield increased with increasing ADFI. Previous studies have shown that improved health conditions, such as increased contents of immunoglobulins and decreased levels of proinflammatory factors, are beneficial to sows' reproductive performance [33]. Moreover, increase in fatty acid content in colostrum and milk also favors the growth performance of lactating piglets [34–36]. Therefore, we can reasonably deduce that the increase in number born alive per litter might be due to the improved health condition of sows fed laminarin-contained diets. Furthermore, the improved growth of lactating piglets might be due to increased sow feed intake by dietary supplementation of laminarin, increasing milk production and enriching nutrients in colostrum and milk.

Laminarin is rich in sulfated polysaccharides and has a positive effect on intestinal health, which may benefit the host by regulating the homeostasis of microbiota [37]. Interactions between microbiota and host cells in the gut are essential for the formation and regulation of the immune system [38]. Proteobacteria is pathogenic to the host, the increased proportion of Proteobacteria reflects the unbalance of intestinal microbiota structure [39]. The aggravation of many diseases is associated with the increased proportion of Proteobacteria [40]. At the phylum level, our study revealed that Proteobacteria in the feces of sows in laminarin group decreased compared with control group. Our study found that Proteobacteria was negatively correlated with the ADFI of lactating sows. Interestingly, Proteobacteria was also negatively correlated with IgM and IgG in colostrum of sows. Based on statistical analysis, the difference in the proportion of *Christensenellaceae_R-7_group* between control and laminarin groups was a significant difference (*P* = 0.01). *Christensenellaceae_R-7_group* was positively correlated with IgG levels in serum and ADFI of sows during lactation, while negatively associated with the contents of IL-2 and progesterone in the serum of sows. *Christensenellaceae_R-7_group* is widely distributed in animal intestine and mucosa, which is very important for host health [41]. Therefore, we supposed that laminarin supplementation might implement its beneficial effect partly through the increase of *Christensenellaceae_R-7_group* abundances in the hindgut of sows during lactation, which warrants continual study.

## Conclusions

In summary, dietary supplementation of laminarin during late pregnancy and lactation significantly increased the milk yield and the ADFI of sows during lactation as well as the number born alive per litter and ADG of suckling piglets, and reduced the culling and death rate of suckling piglets. Laminarin intake reduced the inflammatory response of pregnant sows, and improved the quality of colostrum and milk. Although the diversity of fecal microbiota was not significantly altered, laminarin intake could adjust the composition of hindgut microbiota in which *Christensenellaceae_R-7_group* might play a key role in the improvement of reproductive performance and immune function of sows. Furthermore, this finding can favor the improvement in pregnant nutrition and the healthy status of infants in humans.

### Supplementary Information

**Additional file 1:** **Table S1.** Effects of dietary laminarin supplementation on estradiol, progesterone, leptin, glucose, insulin, and prolactin in serum of sows (*n* = 8–10).** Table S2. **Effects of dietary laminarin supplementation on nutrients composition and immunoglobulins content in milk of sows (*n* = 9). **Table S3. **Effects of dietary laminarin supplementation on amino acids composition in milk of sows (*n* = 9), %.

## Data Availability

Data will be made available on request.

## Abbreviations

ADFI: Average daily feed intake
ADG: Average daily gain
BW: Body weight
BF: Backfat
C10:0: Capric acid
C14:0: Myristic acid
C14:1: Tetradecenoylcarnitine
C16:1: Palmitoleic acid
EPA/C20:5n3: Eicosapentaenoic acid
IL-2: Interleukin-2
IgG: Immunoglobulin G
IFN-γ: Interferon-γ
IL-6: Interleukin-6
IL-10: Interleukin-10
IgA: Immunoglobulin A
IgM: Immunoglobulin M
PUFA: Polyunsaturated fatty acid
SCFAs: Short-chain fatty acids
TNF-α: Tumor necrosis factor-α
