# Supplementary material for: Dietary supplementation of laminarin improves the reproductive performance of sows and the growth of suckling piglets
Source: J Anim Sci Biotechnol. 2023 Sep 10;14:114. doi: 10.1186/s40104-023-00920-6 (PMC10493022; doi:10.1186/s40104-023-00920-6)
Supplement: Supplementary file 1 — Additional file 1: Table S1. Effects of dietary laminarin supplementation on estradiol, progesterone, leptin, glucose, insulin, and prolactin in serum of sows (n = 8–10). Table S2. Effects of dietary laminarin supplementation on nutrients composition and immunoglobulins content in milk of sows (n = 9). Table S3. Effects of dietary laminarin supplementation on amino acids composition in milk of sows (n = 9), %. [file 40104_2023_920_MOESM1_ESM.docx]

**Table S1** Effects of dietary laminarin supplementation on estradiol, progesterone, leptin, glucose, insulin, and prolactin in serum of sows (*n* = 8–10)

| **Items** | **Laminarin levels, %** | | | | **SEM** | ***P-*value** | | |
| --- | --- | --- | --- | --- | --- | --- | --- | --- |
|  | **0** | **0.025** | **0.05** | **0.10** |  |  | **Linear** | **Quadratic** |
| [Estradiol](javascript:;), pg/mL | 18.35 | 21.79 | 16.69 | 17.32 | 1.04 |  | 0.39 | 0.83 |
| Progesterone, ng/mL | 40.70 | 40.23 | 40.46 | 33.59 | 1.10 |  | 0.02 | 0.22 |
| Leptin, ng/mL | 5.30 | 5.72 | 5.68 | 5.46 | 0.10 |  | 0.80 | 0.14 |
| Glucose, mmol/ L | 5.94 | 5.78 | 6.02 | 5.70 | 0.10 |  | 0.51 | 0.63 |
| Insulin, uIU/mL | 15.17 | 14.68 | 14.22 | 14.58 | 0.26 |  | 0.45 | 0.33 |
| Prolactin, uIU/mL | 346.34 | 342.61 | 345.25 | 323.80 | 9.11 |  | 0.39 | 0.70 |

Values are means with SEM

**Table S2** Effects of dietary laminarin supplementation on nutrients composition and immunoglobulins content in milk of sows (*n* = 9)

| **Items** | **Laminarin levels, %** | | **SEM** | ***P*-value** |
| --- | --- | --- | --- | --- |
|  | **0** | **0.10** |  |  |
| Nutrients, % |  |  |  |  |
| Fat | 6.28 | 6.68 | 0.27 | 0.47 |
| Lactose | 4.10 | 3.98 | 0.03 | 0.10 |
| Protein | 6.19 | 5.92 | 0.06 | 0.11 |
| Non-fat solid | 11.20 | 10.87 | 0.10 | 0.11 |
| Density 20 ℃, g/cm^3^ | 1.02 | 0.98 | 0.01 | 0.13 |
| Ash content, % | 0.92 | 0.89 | 0.01 | 0.12 |
| Immunoglobulin, g/L |  |  |  |  |
| IgA | 2.57 | 1.78 | 0.28 | 0.29 |
| IgG | 42.16 | 39.79 | 1.15 | 0.32 |
| IgM | 3.25 | 3.06 | 0.08 | 0.24 |

Values are means with SEM

**Table S3** Effects of dietary laminarin supplementation on amino acids composition in milk of sows (*n* = 9), %

| **Items** | **Laminarin levels, %** | | **SEM** | ***P-*value** |
| --- | --- | --- | --- | --- |
|  | **0** | **0.10** |  |  |
| Asp | 0.42 | 0.41 | 0.007 | 0.45 |
| Thr | 0.21 | 0.21 | 0.004 | 0.69 |
| Ser | 0.26 | 0.26 | 0.005 | 0.99 |
| Glu | 1.05 | 1.06 | 0.014 | 0.91 |
| Pro | 0.56 | 0.57 | 0.009 | 0.59 |
| Gly | 0.16 | 0.16 | 0.002 | 0.65 |
| Ala | 0.18 | 0.18 | 0.004 | 0.61 |
| Cys | 0.07 | 0.07 | 0.002 | 0.65 |
| Val | 0.27 | 0.27 | 0.005 | 0.90 |
| Met | 0.11 | 0.12 | 0.003 | 0.07 |
| Ile | 0.21 | 0.21 | 0.003 | 0.62 |
| Leu | 0.42 | 0.42 | 0.006 | 0.72 |
| Tyr | 0.20 | 0.20 | 0.004 | 0.90 |
| Phe | 0.20 | 0.20 | 0.004 | 0.71 |
| His | 0.14 | 0.15 | 0.003 | 0.46 |
| Lys | 0.38 | 0.39 | 0.006 | 0.51 |
| Arg | 0.24 | 0.24 | 0.005 | 0.48 |
| Trp | 0.06 | 0.06 | 0.001 | 0.85 |

Values are means with SEM
